# Supplementary material for: PERCEPT myeloma: a protocol for a pilot randomised controlled trial of exercise prehabilitation before and during autologous stem cell transplantation in patients with multiple myeloma
Source: BMJ Open. 2020 Jan 29;10(1):e033176. doi: 10.1136/bmjopen-2019-033176 (PMC7044857; doi:10.1136/bmjopen-2019-033176)
Supplement: Author's manuscript [file bmjopen-2019-033176.draft_revisions.pdf]

BMJ Open

BMJ Open is committed to open peer review. As part of this commitment we make the peer review history of every article we publish publicly available.

When an article is published we post the peer reviewers' comments and the authors' responses online. We also post the versions of the paper that were used during peer review. These are the versions that the peer review comments apply to.

The versions of the paper that follow are the versions that were submitted during the peer review process. They are not the versions of record or the final published versions. They should not be cited or distributed as the published version of this manuscript.

BMJ Open is an open access journal and the full, final, typeset and author-corrected version of record of the manuscript is available on our site with no access controls, subscription charges or pay-per-view fees (http://bmjopen.bmj.com).

If you have any questions on BMJ Open's open peer review process please email info.bmjopen@bmj.com

PERCEPT myeloma: a protocol for a pilot randomised controlled trial of exercise prehabilitation before and during autologous stem cell transplantation in patients with myeloma

Journal:	*BMJ Open*
Manuscript ID	bmjopen-2019-033176
Article Type:	Protocol
Date Submitted by the Author:	25-Jul-2019

Complete List of Authors:	McCourt, Orla; University College London Hospitals NHS Foundation Trust, ; University College London, Cancer Institute<br>Fisher, Abigail; University College London, Behavoural Science and Health<br>Ramdharry, Gita; Queen Square Centre for Neuromuscular Diseases<br>Roberts, Anna; University College London, Behavoural Science and Health<br>Land, Joanne; University College London, Behavoural Science and Health<br>Rabin, Neil; University College London Hospitals NHS Foundation Trust, Department of Haematology<br>Yong, Kwee; Univesrsity College London, Research Department of Haematology, Cancer Institute

Keywords:	Hematopoietic Stem Cell Transplantation, Myeloma < HAEMATOLOGY, REHABILITATION MEDICINE, exercise, Physiotherapy

I, the Submitting Author has the right to grant and does grant on behalf of all authors of the Work (as defined in the below author licence), an exclusive licence and/or a non-exclusive licence for contributions from authors who are: i) UK Crown employees; ii) where BMJ has agreed a CC-BY licence shall apply, and/or iii) in accordance with the terms applicable for US Federal Government officers or employees acting as part of their official duties; on a worldwide, perpetual, irrevocable, royalty-free basis to BMJ Publishing Group Ltd ("BMJ") its licensees and where the relevant Journal is co-owned by BMJ to the co-owners of the Journal, to publish the Work in this journal and any other BMJ products and to exploit all rights, as set out in our licence.

The Submitting Author accepts and understands that any supply made under these terms is made by BMJ to the Submitting Author unless you are acting as an employee on behalf of your employer or a postgraduate student of an affiliated institution which is paying any applicable article publishing charge ("APC") for Open Access articles. Where the Submitting Author wishes to make the Work available on an Open Access basis (and intends to pay the relevant APC), the terms of reuse of such Open Access shall be governed by a Creative Commons licence – details of these licences and which Creative Commons licence will apply to this Work are set out in our licence referred to above.

Other than as permitted in any relevant BMJ Author's Self Archiving Policies, I confirm this Work has not been accepted for publication elsewhere, is not being considered for publication elsewhere and does not duplicate material already published. I confirm all authors consent to publication of this Work and authorise the granting of this licence.

For peer review only

TITLE:

PERCEPT myeloma: a protocol for a pilot randomised controlled trial of exercise prehabilitation before and during autologous stem cell transplantation in patients with myeloma

Corresponding Author:

Orla McCourt

Department of Haematology, UCL Cancer Institute, University College London, UK & University College London Hospitals NHS Foundation Trust, London, UK

Postal Address: Therapy Office, Lower Ground Floor (T-1), University College Hospital, 235 Euston Road, London NW1 2BU, UK

Email: o.mccourt@nhs.net

Telephone: 020 7679 1736

Authors:

Orla McCourt – Cancer Institute, University College London, UK & University College London Hospitals NHS Foundation Trust, London, UK

Abigail Fisher - Research Department of Behavioural Science and Health, University College London, London, UK

Gita Ramdharry - Queen Square MRC Centre for Neuromuscular Diseases, Institute of Neurology, University College London, London, UK

Anna Roberts - Research Department of Behavioural Science and Health, University College London, London, UK

Joanne Land - Research Department of Behavioural Science and Health, University College London, London, UK

Neil Rabin – Department of Haematology, University College London Hospitals NHS Foundation Trust, London, UK

Kwee Yong - Cancer Institute, University College London, UK

Word Count: 3604

For peer review only - http://bmjopen.bmj.com/site/about/guidelines.xhtml

ABSTRACT:

Introduction: Myeloma, a blood cancer originating from plasma cells, is the most common indication for autologous stem cell transplantation (SCT). Patients with myeloma undergoing autologous SCT can experience treatment-related morbidity and reduction in function and wellbeing for many months post-treatment. Interventions targeting physical functioning delivered prior to and during SCT have shown promising results in mixed haematological populations and may offer a non-pharmacological solution to physically optimising and preparing myeloma patients for SCT. The aim of this study is to investigate the feasibility of a physiotherapist-led exercise intervention as an integral part of the myeloma autologous SCT pathway at a UK tertiary centre.

Methods & Analysis: A single-site, pilot randomised controlled trial of an exercise intervention embedded within the autologous SCT pathway, compared to usual care, for patients with myeloma. The primary endpoints of this pilot study will be feasibility measures of study and intervention delivery. Secondary endpoints will evaluate any between group differences in cancer-related fatigue, quality of life, functional capacity (six-minute walk test; hand-held dynamometry; a timed sit-to-stand test) and objective and self-reported physical activity and sedentary time. Outcomes will be assessed at four timepoints, prior to SCT, on/around day of SCT, on discharge from SCT hospital admission and 12 weeks post-discharge.

The exercise intervention comprises of partly-supervised aerobic and resistance exercise delivered by a physiotherapist and includes behaviour change techniques to promote adherence and change in exercise behaviour.

The primary outcomes from the trial will be summarised as percentages or mean values with 95% confidence intervals. Between group differences for secondary outcomes at each timepoint will be analysed using appropriate statistic models.

For peer review only - http://bmjopen.bmj.com/site/about/guidelines.xhtml

Ethics & Dissemination: This study has approval from an NHS Health Authority Research Ethics Committee (Camden and Kings Cross, 19/LO/0204). Results will be disseminated through publication in journals and presentations at haematology and rehabilitation-related meetings.

Registration: ISRCTN15875290

KEYWORDS:

Multiple myeloma, Hematopoietic Stem Cell Transplantation, Exercise, Rehabilitation, Physiotherapy, Protocol

ARTICLE SUMMARY

Strengths & Limitations of this study

- This study features a pragmatic trial design integrated into the existing autologous transplant treatment pathway and the use of a partly-supervised exercise intervention, which will incorporate behaviour change techniques, in myeloma patients before, during and after stem cell transplantation.
- It is the first study of a prehabilitation and rehabilitation exercise intervention in myeloma transplant recipients in the UK to incorporate a control arm.
- Although the intervention includes exercise and incorporated behaviour change techniques, it does not include nutritional assessment and/or dietary intervention, as recommended in guidance for prehabilitation interventions.
- The study is a single site pilot trial and is not statistically powered.

For peer review only - http://bmjopen.bmj.com/site/about/guidelines.xhtml

For peer review only

INTRODUCTION

Myeloma is an incurable blood cancer of plasma cells in the bone marrow and accounts for 10% of all haematological cancers[1, 2]. The incidence is approximately 22 cases per 100,000 people in the UK[1]. Although incurable, improved understanding of disease mechanisms and advances in treatments means that survival in myeloma is increasing at the fastest rate among all cancer types (Myeloma UK, 2018). Median estimated survival time for myeloma has quadrupled over the last four decades and 5-year survival is now around 47%[3].

Myeloma patients are treated with autologous stem cell transplantation (SCT) if eligible (based on age and performance status)[4]. SCT involves harvesting the individual's own stem cells and re-infusing them after high dose chemotherapy[5]. Autologous SCT has been shown in multiple randomised controlled trials (RCTs) to prolong progression-free survival, and in some studies, overall survival in myeloma[2, 6]. In the UK, the total numbers of autologous SCTs carried out each year have been increasing by 5% each year[7]. Data from the British Society of Blood and Marrow Transplant (BSBMT) register shows that myeloma is the most common indication for autologous SCT in the UK, with 1411 procedures taking place in 2016[8]. However autologous SCT has a number of side effects[4], including repeated infections, nausea, anorexia, and fatigue and patients can suffer a reduction in their functionality and wellbeing for between six to twelve months post-transplantation[9]. There is a need to develop supportive interventions for patients undergoing SCT aimed at optimising patient fitness before and during SCT in order to mitigate or minimise the negative effects of treatment on physical and psychological outcomes[10]. Prehabilitation and rehabilitation, targeting physical function and fitness, has been suggested as an integral component of myeloma treatment pathways in guidance for managing the consequences of the disease and its treatment, however further research is required to better understand the potential impact of prehabilitation/rehabilitation before and after treatment for myeloma[11].

For peer review only

For peer review only - http://bmjopen.bmj.com/site/about/guidelines.xhtml

There is emerging evidence that exercise during or after SCT may be beneficial. However, existing studies have generally been small, with methodological limitations, particularly the heterogeneity of the interventions and mixed haematological cancer populations studied[12, 13]. Two systematic reviews, of eight and eleven RCTs respectively, have shown that exercise is safe and feasible for people being treated for different types of blood cancer during hospital admission and that the best results are seen when exercise is introduced before or during treatment, when compared to usual care[14, 15].

Only two small trials have explored the use of a pre-transplant exercise intervention in a myeloma only population undergoing autologous SCT, with mixed results[16-18]. The first, a feasibility study of 24 participants showed a significant increase in lean muscle mass in the exercise group[16]. The second study of 166 patients reported a decline in aerobic capacity and increased fatigue in both the intervention and control group, with no difference between groups after SCT. This study, reported in two papers, was also testing the effect of prophylactic epotin alfa (EPO) therapy, a medical product that raises haemoglobin levels, alongside exercise and was therefore not solely assessing the efficacy of the exercise intervention[17, 18]. Additional limitations to these studies include provision of an unsupervised home-based intervention exercise intervention and that the control groups were also advised to walk 20 minutes daily,[16, 17] which may have diluted intervention effects. These studies, however, have demonstrated that it is safe for myeloma patients to exercise whilst undergoing autologous SCT.

Limited published literature exists regarding physical activity (PA) or exercise interventions in patients undergoing SCT originating in the UK. A single arm pilot study of a physiotherapist-led, tailored, exercise intervention delivered to myeloma survivors post-treatment conducted at the authors' centre demonstrated that exercise was associated with improvements in quality of life (QOL), cancer-related fatigue and muscle strength, and was extremely well received by patients[19]. A single-arm

For peer review only

study to test feasibility of delivering exercise during autologous SCT among people with myeloma in a centre in Sheffield has completed recruitment but the results are yet to be published[20].

Objectives

This primary aim of this pilot, single-centre, RCT is to investigate the feasibility of a physiotherapist-led exercise intervention as an integral part of the autologous SCT pathway for patients with myeloma. It is hypothesised that this intervention, delivered before and during autologous SCT treatment for myeloma, will be feasible in terms of recruitment rate and willingness of the participants to be randomised, intervention adherence, compliance to the exercise prescription, and attrition due to the intervention.

Primary objectives of the pilot study are to measure eligibility and recruitment rates from potential participants screened on referral to myeloma outpatient clinics for consideration for autologous SCT; acceptability of the intervention, including adherence to exercise session and deviations from exercise protocol; study completion rate; and any adverse events.

Secondary objectives are to collect preliminary data on the intended primary/secondary outcomes of a future fully-powered RCT, to obtain mean and standard deviation estimates to inform a sample size calculation. These outcomes include: cancer related fatigue; QOL; levels of PA and sedentary time; functional capacity and muscle strength; hospital length of stay, rates of readmission and health service utilisation; and if the trial/exercise intervention is delivered as intended.

Exploratory objectives are to investigate any effect on immune parameters (including immunoglobulins, T cells, B cells, NK cells).

METHODS & ANALYSIS

Participants

For peer review only - http://bmjopen.bmj.com/site/about/guidelines.xhtml

All patients with a diagnosis of myeloma referred to University College London Hospitals NHS Foundation Trust (UCLH) for consideration for autologous SCT will be identified by the myeloma transplant clinical nurse specialists (CNS) and through discussion with the myeloma transplant multidisciplinary team (MDT).

Patient & Public Involvement

The initial aims of this study were developed with input from participants in myeloma survivorship research conducted at our centre. The study has been further developed through involvement of the UCLH Cancer Patient and Public Advisory Panel (CPPAG) during the preparation of the grant application and development of the study protocol. The CPPAG will continue to be provided with updates on the progress of the study and its results.

Setting

UCLH is an acute NHS trust in London, England and tertiary referral centre for SCT. Recruitment and outpatient assessments will take place at the University College Hospital (UCH) Macmillan Cancer Centre. Weekly visits to attend the pre-transplant intervention exercise will take place at UCLH in a gym setting. Exercise and assessments during admission for autologous transplant will take place either in the UCH Macmillan Cancer Centre or on the haematology inpatient wards at UCH.

Recruitment procedures

Potential participants will be screened using the inclusion/exclusion criteria (table 1) by the research team and discussion with the haematology consultants, CNSs and MDT. A participant information sheet (PIS) detailing the study will be provided in a clinical information pack routinely posted to patients prior to their first routine transplant clinic appointment. Potential participants will be contacted by the principle researcher or study physiotherapist, with a follow up telephone call to allow

For peer review only - http://bmjopen.bmj.com/site/about/guidelines.xhtml

patients to ask questions about the study. Those who wish to proceed will be asked to provide informed consent and this will be recorded on the participant consent form. Those approached to take part in the study who are subsequently found to be ineligible or decline to take part will be recorded in a screening log by the principle investigator.

**Inclusion Criteria**	**Exclusion Criteria**
Adult patients with myeloma referred to UCLH for consideration of autologous SCT	Patients with known spinal instability, spinal cord compression or neurological deficits
Clinically able to carry out an exercise training programme on a regular basis	Those who have had recent (within six weeks) spinal surgery or other surgery for pathological fractures
Patients must have a good command of written and spoken English	Abnormal resting ECG and/or unstable angina
Willing and able to provide written informed consent	Those deemed unsuitable to partake by the MDT
	Unable or unwilling to undertake an exercise programme on a regular basis
	Unable or un-willing to provide informed consent

Table 1. Inclusion & Exclusion Criteria

It is conservatively estimated that approximately 60-75 patients will be recruited over a 15-18 month period, with completion of final follow-ups within 18-21 months. Approximately 100 myeloma patients undergo autologous SCT at UCLH each year. As this is a pilot study where the primary

For peer review only - http://bmjopen.bmj.com/site/about/guidelines.xhtml

objective is to evaluate the possibility of providing this intervention as a full trial, there is no plan to replace participants who withdraw.

Randomisation procedures and blinding

Participant randomisation will be undertaken centrally by a researcher not involved in outcome assessment or intervention delivery, using minimisation, with age and gender as the stratification factors using MinimPy, a free, open-source, desktop programme (https://sourceforge.net/projects/minimpy/). This researcher will communicate the allocation of participants to the study physiotherapist who will inform participants of the allocation and deliver the intervention to those in the intervention arm. The principle investigator will attempt to remain blinded to group allocation and will conduct follow-up outcome assessments.

Intervention

The intervention will involve a partly-supervised exercise intervention, incorporating behaviour change techniques, delivered by a physiotherapist and individually tailored to the ability of each participant. The intervention will involve aerobic and resistance exercise during 3 phases of the autologous SCT pathway:

- Phase 1 (6-8 weeks before hospital admission for SCT): Participants will receive one exercise session per week, supervised by a physiotherapist, at a UCLH gym. Participants will be requested to complete two further sessions per week independently.
- Phase 2: (during hospital admission): Immediately after SCT, participants will be offered a supervised exercise session with a physiotherapist in the hospital setting three times per week.

For peer review only - http://bmjopen.bmj.com/site/about/guidelines.xhtml

- Phase 3 (12 weeks after hospital discharge): Participants will be asked to continue exercising independently three times per week and will receive a weekly phone call from a physiotherapist for support and guidance.

Supervised aerobic exercise will comprise of treadmill walking or stationary cycling. Participants will be encouraged to use walking as their aerobic exercise activity, or a stationary bike or elliptical trainer if they have access to one, for independent exercise sessions. In phase 1, prior to transplant admission, aerobic exercise intensity will be targeted between 60-80% of heart rate (HR) reserve (or Karvonen formula), in which Target HR = [(max HR − resting HR) × % intensity] + resting HR, where maximum HR = 220-age[21]. Duration will be started at 15 minutes and progressed by 5 minutes per week to achieve a minimum of 30 minutes by week 3-4. This will be further progressed to 40 minutes by week 5. Target HR will be monitored using HR belts and participants will also use a Rating of Perceived Exertion Scale (RPE)[22]. The participants will be given a scale in their exercise log book, instructed in its use, and advised to work to levels of exertion as determined under supervision. Participants will be encouraged to reach the target duration in one bout but in the presence of discomfort or wish to change exercise machine/method then participants will be required to complete the target duration in bouts of 15 minutes or more.

Resistance exercises will be prescribed to target all major muscle groups (depending on individual health and contraindications). Resistance training will be performed using body weight, progressed with weights or using elastic resistance bands of various strengths. These bands will be supplied to participants at the start of the study to allow convenient independent resistance training. Starting resistance and repetitions of the exercises will be informed by a 10-repetition maximum assessment carried out by the study physiotherapist during the first session. Individually tailoring and gradual progression in resistance training will be prescribed as deemed appropriate at each exercise session by the study physiotherapist according to published principles[23]. Participants will be provided with

For peer review only - http://bmjopen.bmj.com/site/about/guidelines.xhtml

exercise log books and asked to record what exercise they do in the books, as well as document how they feel after exercising and any reasons for not carrying out the exercise as prescribed.

In phase 2, when participants are admitted to hospital for their autologous SCT, they will be offered supervised exercise with the physiotherapist three times per week in the ambulatory care or hospital ward setting (depending on each participant's clinical location), which they can decline if they wish to do so. It is anticipated that participants will be symptomatic from the effects of their autologous SCT, which may include nausea, gastrointestinal complaints and fatigue. They may also experience bleeding and infection as a result of thrombocytopenia and low white cell counts. Therefore the exercise intervention will be highly individualised and tailored according to participant's capacity to carry out the exercises. Aerobic exercise will be carried out, as able, on a stationary bike in bouts of 10 minutes, up to a total duration of 30 minutes. HR will be monitored throughout and participants will be guided to exercise at a RPE (determined by RPE scale) they are comfortable with. Any exercise undertaken, declined or not possible due to clinical indication will be recorded by the physiotherapist in the participants' log book.

In phase 3, when the participants have been discharged from hospital they will be advised by the physiotherapist to continue the independent exercise programme, tailored to their fitness on discharge, until they are 12 weeks post-autologous SCT. During these 12 weeks, they will be contacted once per week via telephone by the physiotherapist to provide support and guidance with continuing to exercise regularly during their recovery and to assist them in progressing their exercise programme. The participants will be encouraged to meet recommended guidance for levels of PA[24] and requested to record any exercise undertaken in their log books.

Previous systematic reviews and meta-analyses have shown that behaviour change techniques (BCTs) from exercise interventions that are associated with improved adherence to exercise by cancer

For peer review only - http://bmjopen.bmj.com/site/about/guidelines.xhtml

survivors include ‘goal setting (behaviour)’, setting ‘graded tasks’, and ‘instruction of how to perform behaviour’,[25, 26] practice and self-monitoring and encouraging participants to attempt to generalise exercise behaviours learned in supervised exercise environments to other, non-supervised contexts[27]. Strategies to promote adherence to the intervention and promote change in exercise behaviour in the current intervention have been coded according to the BCT Taxonomy v1[28]. See Table 2 for incorporated BCTs and examples of how they have been included in the intervention. Study physiotherapists will deliver the intervention and will be provided with a standard operating procedure support the participants to exercise and using BCTs.

Labelled BCT	Component of Intervention
1.1 Goal setting (behaviour)	Overarching goal of exercise programme for all participants is to exercise 3 times per week. Participants also supported to define own personal sub-goals, which are recorded in log book.
1.2 Problem Solving	Enablers, barriers and solutions to barriers completed with physiotherapist and recorded in log book. Problem solving with physiotherapist if discrepancy between current behaviour and goal is identified.
1.4 Action Planning	Attendance at supervised exercise session in gym planned for specific day each week. Participants are also supported to plan independent exercise sessions (e.g. time/day of the week) with physiotherapist. These plans are recorded in log book prior to planned execution.
1.5 Review Behaviour Goal	Participants review performance of exercise sessions recorded in log book against planned goals, and consider modifying goals

For peer review only

For peer review only - http://bmjopen.bmj.com/site/about/guidelines.xhtml

	accordingly with physiotherapist in supervised exercise sessions.
1.6 Discrepancy between current behaviour and goal	Physiotherapist informs participant if there is a discrepancy between current behaviour and goal.
2.2 Feedback on behaviour	Feedback on performance of exercise programme delivered by physiotherapist during supervised exercise sessions (phase 1 and 2) and telephone calls (phase 3).
2.3 Self-monitoring of behaviour	Participants asked to record exercise carried out each week in log book. Heart rate monitoring used in supervised and independent exercise sessions to monitor behaviour being carried out at target intensity.
2.6 Biofeedback	Heart rate monitoring of aerobic exercise effort.
4.1 Instruction on how to perform the behaviour	Supervised exercise sessions with physiotherapist who instructs/teaches participant how to perform behaviour.
5.1 Information about health consequences	Physiotherapist and log book provides information about effects of exercise for health in context of myeloma.
5.4 Monitoring of emotional consequences	Participants encouraged to complete weekly reflections in log book.
5.6 Information about emotional consequences	Physiotherapist and log book provide information about effects of exercise on emotional wellbeing.
6.1 Demonstration of the behaviour	Physiotherapist demonstration and teaching of exercise programme in supervised sessions.
8.1 Behavioural practice/rehearsal	Exercises taught/demonstrated in supervised sessions are practiced within those sessions and independent sessions (phase 1 and 2).

For peer review only

For peer review only - http://bmjopen.bmj.com/site/about/guidelines.xhtml

8.6 Generalisation of target behaviour	Advice to perform exercise programme, learned in supervised session, in independent sessions, during hospital admission and post discharge.
8.7 Graded tasks	Progression of exercise sessions throughout exercise period.
9.1 Credible source	Education and prompting from a physiotherapist with expertise in myeloma/haematology and provided in log book.
9.2 Pros and cons	Prompts to consider advantages and disadvantages of exercising through discussion with physiotherapist and recording in log book.
12.5 Adding objects to the environment	Provision of heart rate monitors, resistance exercise bands and exercise programme sheets/log book for use in independent sessions.
15.1 Verbal persuasion about capability	Verbal support and supervision of physiotherapist to encourage exercise sessions before, during and after ASCT treatment.

Table 2: Behaviour Change Techniques coded to BCT Taxonomy (Michie *et al*., 2013)

For peer review only

Usual care (control) group

Participants randomised to usual care will receive the usual advice provided by haematology CNSs. Participants who specifically ask the research team for exercise advice will be signposted to generic PA advice offered for people undergoing cancer treatment on the Macmillan Cancer support website[29] or from the information and support service within UCLH.

For peer review only - http://bmjopen.bmj.com/site/about/guidelines.xhtml

During hospital admission, patients undergoing SCT are routinely screened by a hospital physiotherapist and receive input for any functional or mobility-related deficits that may prevent or delay hospital discharge. All patients within the study who require physiotherapy or occupational therapy during their hospital admission will receive input as indicated. The indication for and details of input required will be obtained from participants' hospital records and recorded in the study file for enrolled participants who require additional therapy input. Control group participants will not be asked to monitor their activity or receive log books.

Outcome Assessment

The primary endpoints of this pilot study will be study feasibility. A target recruitment rate of >50% of potential participants screened as eligible and approached for this study will be a primary indication of feasibility. Feasibility of delivering the intervention protocol will be assessed by evaluating adherence to the intervention and acceptability of receiving the intervention (including deviations from exercise protocol). Attrition due to the intervention, loss to follow-up and adverse events will also be assessed.

A number of secondary endpoints will also evaluate any between group differences and determine intended primary and secondary endpoints for a fully-powered trial. Fatigue will be assessed using the Functional Assessment of Chronic Illness Therapy (FACIT-F) questionnaire[30]. QOL will be measured with two complementary instruments; the European Organisation for Research and Treatment of Cancer QOL Questionnaire (EORTC QLQ-C30 and myeloma specific module QLQMY20)[31, 32] and the Functional Assessment of Cancer Therapy Bone Marrow Transplantation scale (FACT-BMT) questionnaire[33]. The EORTC QLQ C-30 has a focus on physical and functioning dimensions of QOL whereas FACT-BMT can assess impact of treatment and side-effects upon QOL. Comparison analysis indicates that both instruments should be used together rather than in place of each other when assessing QOL in patients undergoing SCT[34, 35].

For peer review only - http://bmjopen.bmj.com/site/about/guidelines.xhtml

	Screening	Baseline Assessment (T0)	Follow up 1 (T1)	Follow up 2 (T2)	Follow up 3 (T3)
Visit No:		1	2	3	4
	On referral to UCLH ASCT MDT	On day of first transplant clinic appointment	Day of transplant	Day of discharge from hospital	Post-transplant follow-up clinic at 3 months
Qualitative Interview (~20 patients who decline study)	X				
Window of flexibility for timing of visits:		+/-2 days	0+/-1 day	0+/-1 day	+/- 5 days
Screening eligibility	X				
Informed Consent		X			
Demographics & Clinical History		X			
Fatigue: FACT-F		X	X	X	X
QOL: EORTC QLQ C-30-MY20		X	X	X	X

Functional capacity will be assessed with a six-minute walk test (6MWT), hand-held dynamometry and a timed sit to stand test. PA and sedentary time will be recorded over 5-7 days using an ActivPAL accelerometer (PAL Technologies Ltd. Glasgow, UK). Additional measures of self-reported PA behaviour will be captured using the self-complete short form International Physical Activity Questionnaire (IPAQ-SF)[36] and the Exercise Self-Efficacy Scale (ESES)[37]. All these measures will be recorded at baseline and three time points during the autologous SCT pathway.

The outcomes measures and time points for data collection are summarised in table 3.

For peer review only

QOL: FACT-BMT		X	X	X	X
Exercise Behaviour: IPAQ-SF		X	X	X	X
Exercise Self-efficacy Scale		X	X	X	X
Functional Capacity: 6MWT		X	X	X	X
Functional Capacity: 30s STS		X	X	X	X
Functional Capacity: hand held dynamometry		X	X	X	X
Resting BP and HR		X	X	X	X
Height and weight		X	X	X	X
Accelerometery (3-7 days)		X	X	X	X
Qualitative Interview (20 participants)					X
Health and Social Care Service Use: CRSI					X
Blood Counts		X	X	X	X
Levels of Immune Function (blood)		X	X	X	X

Table 3: Study schedule of assessments

For peer review only

Qualitative Data:

Semi-structured interviews will be conducted with a purposeful sample of participants at two time points. Study 'decliners', deemed eligible for the study, who have received a study PIS but declined to enrol in the study will be asked if they would participate in a short interview. These interviews will take place either face to face in the clinic setting or via telephone (based on participants' preference) and will be conducted to explore the patients' experiences of being approached for the exercise study and the process and outcome of their decision-making process in relation to the exercise study.

For peer review only - http://bmjopen.bmj.com/site/about/guidelines.xhtml

Additional semi-structured interviews from approximately 20 participants who enrol and take part in the study will take place at final follow-up, approximately 3 months post-transplant, and will be conducted to explore the participants experiences of autologous SCT treatment and their experiences of being enrolled in the study. Participants will be purposively sampled to include participants from both the intervention arm and the control arm.

Anticipated dates of trial commencement and completion

Recruitment commenced in June 2019, with data collection for all follow-up assessments estimated to be completed by December 2020.

Statistical analysis including sample size calculations

This is a pilot study with no formal power calculation. There is limited available data on the application of an exercise intervention delivered prior to autologous SCT in people with myeloma. This study will provide estimates of means/standard deviations for the intended outcomes of a fully-powered RCT, to support a sample size calculation for a larger trial. For a pilot study, including >30 participants in each arm is considered to be an acceptable number[38, 39].

Data analysis

The primary outcomes (intervention recruitment rate and intervention adherence) from the trial will be summarised as percentages or mean values for each item, with 95% confidence intervals. Data on the acceptability of the randomisation through its effect on dropout rates, completion rates of the outcome assessments and reported use and satisfaction with the intervention materials will also be analysed descriptively. Group differences in fatigue, QOL, functional capacity, PA and sedentary time, self-efficacy at each time point will be analysed using linear regression for continuous factors (e.g.

For peer review only - http://bmjopen.bmj.com/site/about/guidelines.xhtml

For peer review only

fatigue) and frequency tables, chi-squared tests and logistic regression for categorical factors. Gender and age, will be included within the models as covariates. Repeated measures analyses (e.g. mixed models) will be used to analyse the baseline and follow up scores of all measures together. Assessments for each outcome will be made to determine whether the data are normally distributed. For outcomes that are not, even after appropriate transformations, non-parametric methods will be used for data analyses at specific time points.

Qualitative data derived from the interviews of patients will be analysed thematically using an approach that is both deductive and inductive, to ensure that the full range of participants' responses are represented. Codes generated from the data will be assigned to portions of the text to develop an initial coding framework, which will be used by another researcher familiar with the sample population to independently double-code a proportion of the interview transcripts. The framework will be revised to ensure that the codes accurately reflect the data and any discrepancies will be resolved via discussion. The final codes will be applied to the data and incorporated into appropriate themes/sub-themes.

ETHICS & DISSEMINATION

This study has been approved by the NHS Health Authority Research Ethics Committee – Camden and Kings Cross – reference 19/LO/0204. All participants will provide informed written consent to take part and can withdraw at any time.

Results of this study will be written up as part of a doctoral thesis and disseminated through publication in peer reviewed journals, presentation at haematology and rehabilitation-related meetings and shared locally. A lay summary of the study results will be provided to participants who request to receive one after the study have completed.

For peer review only - http://bmjopen.bmj.com/site/about/guidelines.xhtml

DISCUSSION

The study described in this protocol will evaluate the feasibility of carrying out an exercise intervention among people with myeloma before, during and after autologous SCT at a UK centre. There is no standardised or accepted approach to using lead time before SCT to prepare and physically optimise patients and the importance of carrying out research of this kind is particularly pertinent in this population for whom SCT currently offers the best possibility of longer progression-free survival, yet carries the risk of considerable morbidity.

The strengths of this study include the use of a pragmatic intervention using BCTs and designed to integrate into the existing autologous SCT treatment pathway at our centre and the use of a partly-supervised exercise intervention among myeloma patients preparing for transplant. This study is also the first of its kind in the UK to incorporate a control arm. If the feasibility criteria for trial is met and this single site, pilot trial is deemed to be successful it is anticipated to be an easily replicable trial design for future evaluation as a fully-powered multicentre trial. Recent guidance for prehabilitation in cancer advocates for a multimodal intervention, comprising of exercise, nutrition and psychological/behaviour change assessment and support[40]. There is no nutritional assessment or dietary intervention included in this study which may be considered a limitation of this exercise focussed intervention. It is expected that this pilot study will be acceptable and feasible and an important step in developing the field of exercise prehabilitation and rehabilitation within haematological cancer treatment in the UK. Dissemination of study results is expected in early 2021.

AUTHORS CONTRIBUTIONS

OM drafted the manuscript. OM, KY, AF, GR designed the study and secured funding and ethical approval. AF, JL, NR provided intellectual input to the final study protocol according to their area of expertise. All authors contributed to revising and final approval of the submitted manuscript.

For peer review only - http://bmjopen.bmj.com/site/about/guidelines.xhtml

FUNDING STATEMENT

This report is independent research supported by the National Institute for Health Research (HEE/ NIHR ICA Programme Clinical Doctoral Research Fellowship, Ms Orla McCourt, ICA-CDRF-2017-03-067). The views expressed in this publication are those of the author(s) and not necessarily those of the NHS, the National Institute for Health Research or the Department of Health and Social Care.

COMPETING INTERESTS STATEMENT

None

References:

1. Haematological Malignancy Research Network (HMRN). Prevalence 2019. Available from: https://www.hmrn.org/statistics/prevalence [Accessed June 2019].
2. Gozzetti A, Candi V, Papini G, et al. Therapeutic Advancements in Multiple Myeloma. Frontiers in Oncology. 2014;4:241.
3. Cancer Research UK. Myeloma Statistics 2015. Available from: http://www.cancerresearchuk.org/health-professional/cancer-statistics/statistics-by-cancer-type/myeloma#heading-Zero [Accessed June 2019].
4. Mahajan S, Tandon N, Kumar S. The evolution of stem-cell transplantation in multiple myeloma. Ther Adv Hematol. 2018;9(5):123-33.
5. NHS Commissioning Board. Clinical Commissioning Policy: Haematopoietic Stem Cell Transplantation England: NHS Commissioning Board; 2013.
6. Krejci M, Scudla V, Tothova E, et al. Long-term outcomes of autologous transplantation in multiple myeloma: significant survival benefit of novel drugs in post-transplantation relapse. Clin Lymphoma Myeloma. 2009;9(6):436-42.
7. NHS England. Clinical Commissioning Policy: Haematolopoietic Stem Cell Transplantation (HSCT): Revised (All Ages). In: England N, editor. England2015.
8. British Society of Blood and Marrow Transplantation. 2016 Activity 2016. Available from: http://www.bsbmt.org/activity/2016/ [Accessed June 2019].
9. Pidala J, Anasetti C, Jim H. Health-related quality of life following haematopoietic cell transplantation: patient education, evaluation and intervention. Br J Haematol. 2010;148(3):373-85.
10. Ahmedzai SH, Snowden JA, Ashcroft AJ, et al. Patient-Reported Outcome Results From the Open-Label, Randomized Phase III Myeloma X Trial Evaluating Salvage Autologous Stem-Cell Transplantation in Relapsed Multiple Myeloma. Journal of clinical oncology : official journal of the American Society of Clinical Oncology. 2019:Jco1801006.

For peer review only - http://bmjopen.bmj.com/site/about/guidelines.xhtml

For peer review only

11. Snowden JA, Greenfield DM, Bird JM, et al. Guidelines for screening and management of late and long-term consequences of myeloma and its treatment. Br J Haematol. 2017;176(6):888-907.
12. Liu RD, Chinapaw MJ, Huijgens PC, et al. Physical exercise interventions in haematological cancer patients, feasible to conduct but effectiveness to be established: a systematic literature review. Cancer Treat Rev. 2009;35(2):185-92.
13. Wiskemann J, Huber G. Physical exercise as adjuvant therapy for patients undergoing hematopoietic stem cell transplantation. Bone marrow transplantation. 2008;41(4):321-9.
14. Persoon S, Kersten MJ, van der Weiden K, et al. Effects of exercise in patients treated with stem cell transplantation for a hematologic malignancy: a systematic review and meta-analysis. Cancer Treat Rev. 2013;39(6):682-90.
15. van Haren IE, Timmerman H, Potting CM, et al. Physical exercise for patients undergoing hematopoietic stem cell transplantation: systematic review and meta-analyses of randomized controlled trials. Phys Ther. 2013;93(4):514-28.
16. Coleman EA, Coon S, Hall-Barrow J, et al. Feasibility of exercise during treatment for multiple myeloma. Cancer nursing. 2003;26(5):410-9.
17. Coleman EA, Coon SK, Kennedy RL, et al. Effects of exercise in combination with epoetin alfa during high-dose chemotherapy and autologous peripheral blood stem cell transplantation for multiple myeloma. Oncol Nurs Forum. 2008;35(3):E53-61.
18. Coleman EA, Goodwin JA, Kennedy R, et al. Effects of exercise on fatigue, sleep, and performance: a randomized trial. Oncology nursing forum. 2012;39(5):468-77.
19. Groeneveldt L, Mein G, Garrod R, et al. A mixed exercise training programme is feasible and safe and may improve quality of life and muscle strength in multiple myeloma survivors. BMC cancer. 2013;13:31.
20. Keen C, Skilbeck J, Ross H, et al. Is it feasible to conduct a randomised controlled trial of pretransplant exercise (prehabilitation) for patients with multiple myeloma awaiting autologous haematopoietic stem cell transplantation? Protocol for the PREeMPT study. BMJ Open. 2018;8(3).
21. Karvonen MJ, Kentala E, Mustala O. The effects of training on heart rate; a longitudinal study. Annales medicinae experimentalis et biologiae Fenniae. 1957;35(3):307-15.
22. Borg GA. Psychophysical bases of perceived exertion. Med Sci Sports Exerc. 1982;14(5):377-81.
23. Kraemer WJ, Ratamess NA. Fundamentals of resistance training: progression and exercise prescription. Med Sci Sports Exerc. 2004;36(4):674-88.
24. Department of Health & Social Care. Start active, stay active: a report of physical activity from the four home countries Chief Medical Officers. 2011.
25. Bourke L, Homer KE, Thaha MA, et al. Interventions for promoting habitual exercise in people living with and beyond cancer. Cochrane Database Syst Rev. 2013(9):CD010192.
26. Turner RR, Steed L, Quirk H, et al. Interventions for promoting habitual exercise in people living with and beyond cancer. Cochrane Database Syst Rev. 2018;9:Cd010192.
27. Hobbs N, Godfrey A, Lara J, et al. Are behavioral interventions effective in increasing physical activity at 12 to 36 months in adults aged 55 to 70 years? a systematic review and meta-analysis. BMC Medicine. 2013;11(1):75.
28. Michie S, Richardson M, Johnston M, et al. The behavior change technique taxonomy (v1) of 93 hierarchically clustered techniques: building an international consensus for the reporting of behavior change interventions. Ann Behav Med. 2013;46(1):81-95.
29. Macmillan Cancer Support. Physical Activity Resources 2019. Available from: https://www.macmillan.org.uk/about-us/health-professionals/programmes-and-services/physical-activity/resources.html [Accessed July 2019].
30. Webster K, Cella D, Yost K. The Functional Assessment of Chronic Illness Therapy (FACIT) Measurement System: properties, applications, and interpretation. Health Qual Life Outcomes. 2003;1:79.

For peer review only - http://bmjopen.bmj.com/site/about/guidelines.xhtml

31. Aaronson NK, Ahmedzai S, Bergman B, et al. The European Organization for Research and Treatment of Cancer QLQ-C30: a quality-of-life instrument for use in international clinical trials in oncology. J Natl Cancer Inst. 1993;85(5):365-76.
32. Cocks K, Cohen D, Wisloff F, et al. An international field study of the reliability and validity of a disease-specific questionnaire module (the QLQ-MY20) in assessing the quality of life of patients with multiple myeloma. Eur J Cancer. 2007;43(11):1670-8.
33. McQuellon RP, Russell GB, Cella DF, et al. Quality of life measurement in bone marrow transplantation: development of the Functional Assessment of Cancer Therapy-Bone Marrow Transplant (FACT-BMT) scale. Bone marrow transplantation. 1997;19(4):357-68.
34. Grulke N, Albani C, Bailer H. Quality of life in patients before and after haematopoietic stem cell transplantation measured with the European Organization for Research and Treatment of Cancer (EORTC) Quality of Life Core Questionnaire QLQ-C30. Bone marrow transplantation. 2012;47(4):473-82.
35. Kopp M, Schweigkofler H, Holzner B, et al. EORTC QLQ-C30 and FACT-BMT for the measurement of quality of life in bone marrow transplant recipients: a comparison. European journal of haematology. 2000;65(2):97-103.
36. Craig CL, Marshall AL, Sjostrom M, et al. International physical activity questionnaire: 12-country reliability and validity. Med Sci Sports Exerc. 2003;35(8):1381-95.
37. Resnick B, Jenkins LS. Testing the reliability and validity of the Self-Efficacy for Exercise scale. Nurs Res. 2000;49(3):154-9.
38. Lancaster GA, Dodd S, Williamson PR. Design and analysis of pilot studies: recommendations for good practice. J Eval Clin Pract. 2004;10(2):307-12.
39. Browne RH. On the use of a pilot sample for sample size determination. Stat Med. 1995;14(17):1933-40.
40. Macmillan Cancer Support. Prehabilitation for people with cancer: Principles and guidance for prehabilitation within the management and support of people with cancer. England; 2019.

For peer review only

For peer review only - http://bmjopen.bmj.com/site/about/guidelines.xhtml

SPIRIT

STANDARD PROTOCOL ITEMS: RECOMMENDATIONS FOR INTERVENTIONAL TRIALS

SPIRIT 2013 Checklist: Recommended items to address in a clinical trial protocol and related documents*

Section/item	Item No	Description
**Administrative information**		
Title	1	Descriptive title identifying the study design, population, interventions, and, if applicable, trial acronym
Trial registration	2a	Trial identifier and registry name. If not yet registered, name of intended registry
	2b	All items from the World Health Organization Trial Registration Data Set
Protocol version	3	Date and version identifier
Funding	4	Sources and types of financial, material, and other support
Roles and responsibilities	5a	Names, affiliations, and roles of protocol contributors
	5b	Name and contact information for the trial sponsor
	5c	Role of study sponsor and funders, if any, in study design; collection, management, analysis, and interpretation of data; writing of the report; and the decision to submit the report for publication, including whether they will have ultimate authority over any of these activities
	5d	Composition, roles, and responsibilities of the coordinating centre, steering committee, endpoint adjudication committee, data management team, and other individuals or groups overseeing the trial, if applicable (see Item 21a for data monitoring committee)
**Introduction**		
Background and rationale	6a	Description of research question and justification for undertaking the trial, including summary of relevant studies (published and unpublished) examining benefits and harms for each intervention
	6b	Explanation for choice of comparators
Objectives	7	Specific objectives or hypotheses
Trial design	8	Description of trial design including type of trial (eg, parallel group, crossover, factorial, single group), allocation ratio, and framework (eg, superiority, equivalence, noninferiority, exploratory)

For peer review only

Methods: Participants, interventions, and outcomes

Study setting	9	Description of study settings (eg, community clinic, academic hospital) and list of countries where data will be collected. Reference to where list of study sites can be obtained
Eligibility criteria	10	Inclusion and exclusion criteria for participants. If applicable, eligibility criteria for study centres and individuals who will perform the interventions (eg, surgeons, psychotherapists)
Interventions	11a	Interventions for each group with sufficient detail to allow replication, including how and when they will be administered
	11b	Criteria for discontinuing or modifying allocated interventions for a given trial participant (eg, drug dose change in response to harms, participant request, or improving/worsening disease)
	11c	Strategies to improve adherence to intervention protocols, and any procedures for monitoring adherence (eg, drug tablet return, laboratory tests)
	11d	Relevant concomitant care and interventions that are permitted or prohibited during the trial
Outcomes	12	Primary, secondary, and other outcomes, including the specific measurement variable (eg, systolic blood pressure), analysis metric (eg, change from baseline, final value, time to event), method of aggregation (eg, median, proportion), and time point for each outcome. Explanation of the clinical relevance of chosen efficacy and harm outcomes is strongly recommended
Participant timeline	13	Time schedule of enrolment, interventions (including any run-ins and washouts), assessments, and visits for participants. A schematic diagram is highly recommended (see Figure)
Sample size	14	Estimated number of participants needed to achieve study objectives and how it was determined, including clinical and statistical assumptions supporting any sample size calculations
Recruitment	15	Strategies for achieving adequate participant enrolment to reach target sample size

Methods: Assignment of interventions (for controlled trials)

Allocation:

Sequence generation	16a	Method of generating the allocation sequence (eg, computer-generated random numbers), and list of any factors for stratification. To reduce predictability of a random sequence, details of any planned restriction (eg, blocking) should be provided in a separate document that is unavailable to those who enrol participants or assign interventions

Allocation concealment mechanism	16b	Mechanism of implementing the allocation sequence (eg, central telephone; sequentially numbered, opaque, sealed envelopes), describing any steps to conceal the sequence until interventions are assigned
Implementation	16c	Who will generate the allocation sequence, who will enrol participants, and who will assign participants to interventions
Blinding (masking)	17a	Who will be blinded after assignment to interventions (eg, trial participants, care providers, outcome assessors, data analysts), and how
	17b	If blinded, circumstances under which unblinding is permissible, and procedure for revealing a participant's allocated intervention during the trial
**Methods: Data collection, management, and analysis**		
Data collection methods	18a	Plans for assessment and collection of outcome, baseline, and other trial data, including any related processes to promote data quality (eg, duplicate measurements, training of assessors) and a description of study instruments (eg, questionnaires, laboratory tests) along with their reliability and validity, if known. Reference to where data collection forms can be found, if not in the protocol
	18b	Plans to promote participant retention and complete follow-up, including list of any outcome data to be collected for participants who discontinue or deviate from intervention protocols
Data management	19	Plans for data entry, coding, security, and storage, including any related processes to promote data quality (eg, double data entry; range checks for data values). Reference to where details of data management procedures can be found, if not in the protocol
Statistical methods	20a	Statistical methods for analysing primary and secondary outcomes. Reference to where other details of the statistical analysis plan can be found, if not in the protocol
	20b	Methods for any additional analyses (eg, subgroup and adjusted analyses)
	20c	Definition of analysis population relating to protocol non-adherence (eg, as randomised analysis), and any statistical methods to handle missing data (eg, multiple imputation)
**Methods: Monitoring**		
Data monitoring	21a	Composition of data monitoring committee (DMC); summary of its role and reporting structure; statement of whether it is independent from the sponsor and competing interests; and reference to where further details about its charter can be found, if not in the protocol. Alternatively, an explanation of why a DMC is not needed

	21b	Description of any interim analyses and stopping guidelines, including who will have access to these interim results and make the final decision to terminate the trial
Harms	22	Plans for collecting, assessing, reporting, and managing solicited and spontaneously reported adverse events and other unintended effects of trial interventions or trial conduct
Auditing	23	Frequency and procedures for auditing trial conduct, if any, and whether the process will be independent from investigators and the sponsor
**Ethics and dissemination**		
Research ethics approval	24	Plans for seeking research ethics committee/institutional review board (REC/IRB) approval
Protocol amendments	25	Plans for communicating important protocol modifications (eg, changes to eligibility criteria, outcomes, analyses) to relevant parties (eg, investigators, REC/IRBs, trial participants, trial registries, journals, regulators)
Consent or assent	26a	Who will obtain informed consent or assent from potential trial participants or authorised surrogates, and how (see Item 32)
	26b	Additional consent provisions for collection and use of participant data and biological specimens in ancillary studies, if applicable
Confidentiality	27	How personal information about potential and enrolled participants will be collected, shared, and maintained in order to protect confidentiality before, during, and after the trial
Declaration of interests	28	Financial and other competing interests for principal investigators for the overall trial and each study site
Access to data	29	Statement of who will have access to the final trial dataset, and disclosure of contractual agreements that limit such access for investigators
Ancillary and post-trial care	30	Provisions, if any, for ancillary and post-trial care, and for compensation to those who suffer harm from trial participation
Dissemination policy	31a	Plans for investigators and sponsor to communicate trial results to participants, healthcare professionals, the public, and other relevant groups (eg, via publication, reporting in results databases, or other data sharing arrangements), including any publication restrictions
	31b	Authorship eligibility guidelines and any intended use of professional writers
	31c	Plans, if any, for granting public access to the full protocol, participant-level dataset, and statistical code

For peer review only

Appendices

Informed consent materials	32	Model consent form and other related documentation given to participants and authorised surrogates
Biological specimens	33	Plans for collection, laboratory evaluation, and storage of biological specimens for genetic or molecular analysis in the current trial and for future use in ancillary studies, if applicable

*It is strongly recommended that this checklist be read in conjunction with the SPIRIT 2013 Explanation & Elaboration for important clarification on the items. Amendments to the protocol should be tracked and dated. The SPIRIT checklist is copyrighted by the SPIRIT Group under the Creative Commons "Attribution-NonCommercial-NoDerivs 3.0 Unported" license.

For peer review only

BMJ Open

PERCEPT myeloma: a protocol for a pilot randomised controlled trial of exercise prehabilitation before and during autologous stem cell transplantation in patients with multiple myeloma

Journal:	*BMJ Open*
Manuscript ID	bmjopen-2019-033176.R1
Article Type:	Protocol
Date Submitted by the Author:	13-Dec-2019
Complete List of Authors:	McCourt, Orla; University College London Hospitals NHS Foundation Trust, ; University College London, Cancer Institute<br>Fisher, Abigail; University College London, Behavoural Science and Health<br>Ramdharry, Gita; Queen Square Centre for Neuromuscular Diseases<br>Roberts, Anna; University College London, Behavoural Science and Health<br>Land, Joanne; University College London, Behavoural Science and Health<br>Rabin, Neil; University College London Hospitals NHS Foundation Trust, Department of Haematology<br>Yong, Kwee; Univesrsity College London, Research Department of Haematology, Cancer Institute
**Primary Subject Heading**:	Haematology (incl blood transfusion)
Secondary Subject Heading:	Rehabilitation medicine
Keywords:	Hematopoietic Stem Cell Transplantation, Myeloma < HAEMATOLOGY, REHABILITATION MEDICINE, exercise, Physiotherapy

SCHOLARONE™
Manuscripts

I, the Submitting Author has the right to grant and does grant on behalf of all authors of the Work (as defined in the below author licence), an exclusive licence and/or a non-exclusive licence for contributions from authors who are: i) UK Crown employees; ii) where BMJ has agreed a CC-BY licence shall apply, and/or iii) in accordance with the terms applicable for US Federal Government officers or employees acting as part of their official duties; on a worldwide, perpetual, irrevocable, royalty-free basis to BMJ Publishing Group Ltd ("BMJ") its licensees and where the relevant Journal is co-owned by BMJ to the co-owners of the Journal, to publish the Work in this journal and any other BMJ products and to exploit all rights, as set out in our licence.

The Submitting Author accepts and understands that any supply made under these terms is made by BMJ to the Submitting Author unless you are acting as an employee on behalf of your employer or a postgraduate student of an affiliated institution which is paying any applicable article publishing charge ("APC") for Open Access articles. Where the Submitting Author wishes to make the Work available on an Open Access basis (and intends to pay the relevant APC), the terms of reuse of such Open Access shall be governed by a Creative Commons licence – details of these licences and which Creative Commons licence will apply to this Work are set out in our licence referred to above.

Other than as permitted in any relevant BMJ Author's Self Archiving Policies, I confirm this Work has not been accepted for publication elsewhere, is not being considered for publication elsewhere and does not duplicate material already published. I confirm all authors consent to publication of this Work and authorise the granting of this licence.

For peer review only

TITLE:

PERCEPT myeloma: a protocol for a pilot randomised controlled trial of exercise prehabilitation before and during autologous stem cell transplantation in patients with multiple myeloma

Corresponding Author:

Orla McCourt

Department of Haematology, UCL Cancer Institute, University College London, UK & University College London Hospitals NHS Foundation Trust, London, UK

Postal Address: Therapy Office, Lower Ground Floor (T-1), University College Hospital, 235 Euston Road, London NW1 2BU, UK

Email: o.mccourt@nhs.net

Telephone: 020 7679 1736

Authors:

Orla McCourt – Cancer Institute, University College London, UK & University College London Hospitals NHS Foundation Trust, London, UK

Abigail Fisher - Research Department of Behavioural Science and Health, University College London, London, UK

Gita Ramdharry - Queen Square MRC Centre for Neuromuscular Diseases, Institute of Neurology, University College London, London, UK

Anna Roberts - Research Department of Behavioural Science and Health, University College London, London, UK

Joanne Land - Research Department of Behavioural Science and Health, University College London, London, UK

Neil Rabin – Department of Haematology, University College London Hospitals NHS Foundation Trust, London, UK

Kwee Yong - Cancer Institute, University College London, UK

Word Count: 3952

For peer review only - http://bmjopen.bmj.com/site/about/guidelines.xhtml

ABSTRACT:

Introduction: Myeloma, a blood cancer originating from plasma cells, is the most common indication for autologous stem cell transplantation (SCT). Patients with myeloma undergoing autologous SCT experience treatment-related morbidity and reduction in function and wellbeing for many months post-treatment. Interventions targeting physical functioning delivered prior to and during SCT have shown promising results in mixed haematological populations and may offer a non-pharmacological solution to physically optimising and preparing patients for SCT. The aim of this study is to investigate the feasibility of a physiotherapist-led exercise intervention as an integral part of the myeloma autologous SCT pathway at a UK tertiary centre.

Methods & Analysis: PERCEPT is a single-site, pilot randomised controlled trial of an exercise intervention embedded within the myeloma autologous SCT pathway, compared to usual care. The primary study endpoints will be feasibility measures of study and intervention delivery including recruitment rates, acceptability of intervention, study completion rate and any adverse events. Secondary endpoints will evaluate differences between the exercise intervention group and the usual care control group in cancer-related fatigue, quality of life, functional capacity (six-minute walk test; hand-held dynamometry; a timed sit-to-stand test) and objective and self-reported physical activity. Outcomes will be assessed at four timepoints, approximately 6-8 weeks prior to SCT, on/around day of SCT, on discharge from SCT hospital admission and 12 weeks post-discharge.

The exercise intervention comprises of partly-supervised physiotherapist-led aerobic and resistance exercise including behaviour change techniques to promote change in exercise behaviour.

The primary outcomes from the trial will be summarised as percentages or mean values with 95% confidence intervals. Between group differences for secondary outcomes at each timepoint will be analysed using appropriate statistic models.

For peer review only - http://bmjopen.bmj.com/site/about/guidelines.xhtml

Ethics & Dissemination: This study has NHS REC approval (Camden and Kings Cross, 19/LO/0204). Results will be disseminated through publication and presentations at haematology and rehabilitation-related meetings.

Registration: ISRCTN15875290

KEYWORDS:

Multiple myeloma, Hematopoietic Stem Cell Transplantation, Exercise, Rehabilitation, Physiotherapy, Protocol

ARTICLE SUMMARY

Strengths & Limitations of this study

- This study features a pragmatic trial design integrated into the existing autologous transplant treatment pathway and the use of a partly-supervised exercise intervention, which will incorporate behaviour change techniques, in myeloma patients before, during and after stem cell transplantation.
- It is the first study of a prehabilitation and rehabilitation exercise intervention in myeloma transplant recipients in the UK to incorporate a control arm.
- Although the intervention includes exercise and incorporated behaviour change techniques, it does not include nutritional assessment and/or dietary intervention, as recommended in guidance for prehabilitation interventions.
- The study is a single site pilot trial and is not statistically powered.

For peer review only - http://bmjopen.bmj.com/site/about/guidelines.xhtml

INTRODUCTION

Myeloma, also known as multiple myeloma, is an incurable blood cancer of plasma cells in the bone marrow and accounts for 10% of all haematological cancers[1,2]. The incidence is approximately 22 cases per 100,000 people in the UK[1]. Although incurable, improved understanding of disease mechanisms and advances in treatments means that survival in myeloma is increasing at the fastest rate among all cancer types[3].Median estimated survival time for myeloma has quadrupled over the last four decades and 5-year survival is now around 47%[4].

Myeloma patients are treated with autologous stem cell transplantation (SCT) if eligible (based on age and performance status)[5]. Autologous SCT (ASCT) involves harvesting the individual's own stem cells and re-infusing them after high dose chemotherapy[6]. ASCT has been shown in multiple randomised controlled trials (RCTs) to prolong progression-free survival, and in some studies, overall survival in myeloma[2,7]. In the UK, the total numbers of ASCTs carried out each year have been increasing by 5% each year[8]. Data from the British Society of Blood and Marrow Transplant (BSBMT) register shows that myeloma is the most common indication for ASCT in the UK, with 1411 procedures taking place in 2016[9]. However ASCT has a number of side effects[5], including repeated infections, nausea, anorexia, and fatigue and patients can suffer a reduction in their functionality and wellbeing for between six to twelve months post-transplantation[10]. There is a need to develop supportive interventions for patients undergoing ASCT aimed at optimising patient fitness before and during ASCT in order to mitigate or minimise the negative effects of treatment on physical and psychological outcomes[11]. Prehabilitation and rehabilitation, targeting physical function and fitness, has been suggested as an integral component of myeloma treatment pathways in guidance for managing the consequences of the disease and its treatment, however further research is required to better understand the potential impact of prehabilitation/rehabilitation before and after treatment for myeloma[12]. Given the prevalence of myeloma-related bone destruction in this population, it is also vital to develop and test tailored exercise interventions that are safe and effective at enhancing

For peer review only - http://bmjopen.bmj.com/site/about/guidelines.xhtml

physical capacity in the presence of disease-related skeletal deformity and pain, common symptoms that limit patients with myeloma being physically active during and after treatment[13].

There is emerging evidence that exercise during or after SCT may be beneficial. However, existing studies have generally been small, with methodological limitations, particularly the heterogeneity of the interventions and mixed haematological cancer populations studied[14,15]. Two systematic reviews, of eight and eleven RCTs respectively, have shown that exercise is safe and feasible for people being treated for different types of blood cancer during hospital admission and that the best results are seen when exercise is introduced before or during treatment, when compared to usual care. The majority of the studies included in these reviews were carried out with patients undergoing allogeneic SCT or mixed samples of both allogeneic and autologous SCT recipients using varied interventions [16,17]. There is acknowledged variation in symptoms and consequences of treatment experienced by patients with different bloods cancers, and between undergoing allogeneic and autologous SCT[16,18]. Studies among allogeneic SCT recipients highlight the intensive nature of the medical intervention and its associated symptom burden and consequences, most notably graft versus host disease (GvHD) and an increased risk of treatment associated mortality[19]. In contrast, with autologous transplantation the mortality is considerably lower and there is no risk of GvHD, with its attendant risk of infections and short and long term morbidity[18]. These variations in clinical features support the need to study rehabilitation interventions in uniform blood cancer patient populations.

Only two small trials have explored the use of a pre-transplant exercise in a myeloma only population undergoing ASCT, with mixed results[20-22]. The first, a feasibility study of 24 participants showed a significant increase in lean muscle mass in the exercise group[20]. The second study of 166 patients reported a decline in aerobic capacity and increased fatigue in both the experimental and control group, with no difference between groups after SCT. This study, reported in two papers, was also testing the effect of prophylactic epotin alfa (EPO) therapy, a medical product that raises haemoglobin levels, alongside exercise and was therefore not solely assessing the efficacy of the prescribed exercise

For peer review only - http://bmjopen.bmj.com/site/about/guidelines.xhtml

[21,22]. Additional limitations to these studies include provision of an unsupervised home-based exercise intervention and that the control groups were also advised to walk 20 minutes daily,[20,21] which may have diluted the effect size. These studies, however, have demonstrated that it is safe for myeloma patients to exercise whilst undergoing ASCT.

Limited published literature exists regarding physical activity (PA) or exercise interventions in patients undergoing SCT originating in the UK. A single arm pilot study of a physiotherapist-led, tailored, exercise intervention delivered to myeloma survivors post-treatment conducted at the authors' centre demonstrated that exercise was associated with improvements in quality of life (QOL), cancer-related fatigue and muscle strength, and was extremely well received by patients[23]. A single-arm study to test feasibility of delivering exercise during ASCT among people with myeloma in a centre in Sheffield has completed recruitment but the results are yet to be published[24].

Objectives

This primary aim of this pilot, single-centre, RCT is to investigate the feasibility of a physiotherapist-led exercise intervention as an integral part of the ASCT pathway for patients with myeloma. It is hypothesised that this intervention, delivered before and during ASCT treatment for myeloma, will be feasible in terms of recruitment rate and willingness of the participants to be randomised, intervention adherence, compliance to the exercise prescription, and attrition due to the intervention.

Primary objectives of the pilot study are to measure eligibility and recruitment rates from potential participants screened on referral to myeloma outpatient clinics for consideration for ASCT; acceptability of the intervention, including adherence to exercise session and deviations from exercise protocol; study completion rate; and any adverse events.

Secondary objectives are to collect preliminary data on the intended primary/secondary outcomes of a future fully-powered RCT, to obtain mean and standard deviation estimates to inform a sample size

For peer review only - http://bmjopen.bmj.com/site/about/guidelines.xhtml

calculation. These outcomes include: cancer related fatigue; QOL; levels of PA and sedentary time; functional capacity and muscle strength; hospital length of stay, rates of readmission and health service utilisation; and if the trial/exercise intervention is delivered as intended.

Exploratory objectives are to investigate any effect on immune parameters (including immunoglobulins, T cells, B cells, NK cells).

METHODS & ANALYSIS

Participants

All patients with a diagnosis of myeloma referred to University College London Hospitals NHS Foundation Trust (UCLH) for consideration for ASCT will be identified through discussion with the clinical multidisciplinary team (MDT) responsible for coordinating treatment for patients with myeloma, which includes the medical team and clinical nurse specialists (CNSs), nurses who act in a key working role to provide support for patients undergoing cancer treatment[25].

Inclusion and Exclusion Criteria

Potential participants will be screened using the inclusion/exclusion criteria by the research team and through discussion with the haematology consultants, CNSs and MDT. All adult patients with myeloma referred to UCLH for consideration of ASCT, who are clinically able to carry out an exercise training programme on a regular basis and willing and able to provide written informed consent will be deemed eligible to approach. Patients must have a good command of written and spoken English.

Patients will be excluded if they have known spinal instability, spinal cord compression or neurological deficits, have had recent (within six weeks) spinal surgery or other surgery for pathological fractures, have abnormal resting ECG and/or unstable angina. Patients will also be excluded if they are unable or unwilling to undertake an exercise programme on a regular basis or are unable or un-willing to provide informed consent.

For peer review only - http://bmjopen.bmj.com/site/about/guidelines.xhtml

Patient & Public Involvement

The initial aims of this study were developed with input from participants in myeloma survivorship research conducted at our centre. The study has been further developed through involvement of the UCLH Cancer Patient and Public Advisory Panel (CPPAG) during the preparation of the grant application and development of the study protocol. The CPPAG will continue to be provided with updates on the progress of the study and its results.

Setting

UCLH is an acute NHS trust in London, England and tertiary referral centre for SCT. Recruitment and outpatient assessments will take place at the University College Hospital (UCH) Macmillan Cancer Centre. Weekly visits to attend the pre-transplant intervention exercise will take place at UCLH in a gym setting. Exercise and assessments during admission for autologous transplant will take place either in the UCH Macmillan Cancer Centre or on the haematology inpatient wards at UCH.

Recruitment procedures

A participant information sheet (PIS) detailing the study will be provided in a clinical information pack routinely posted to patients prior to their first routine transplant clinic appointment. Potential participants will be contacted by the principle researcher or study physiotherapist, with a follow up telephone call to allow patients to ask questions about the study. Those who wish to proceed will be asked to provide informed consent and this will be recorded on the participant consent form. Those approached to take part in the study who are subsequently found to be ineligible or decline to take part will be recorded in a screening log by the principle investigator.

For peer review only - http://bmjopen.bmj.com/site/about/guidelines.xhtml

For peer review only

It is conservatively estimated that approximately 60-75 patients will be recruited over a 15-18 month period, with completion of final follow-ups within 18-21 months. Approximately 100 myeloma patients undergo ASCT at UCLH each year.

Randomisation procedures and blinding

Participant randomisation will be undertaken centrally by a researcher not involved in outcome assessment or intervention delivery, using minimisation, with age and gender as the stratification factors using MinimPy, a free, open-source, desktop programme (https://sourceforge.net/projects/minimpy/). This researcher will communicate the allocation of participants to the physiotherapist who will inform participants of the allocation and deliver the intervention to those in the intervention arm. The principle investigator will attempt to remain blinded to group allocation and will conduct follow-up outcome assessments.

Intervention

The intervention will involve a partly-supervised exercise intervention, incorporating behaviour change techniques, delivered by a physiotherapist and individually tailored to the ability of each participant. The intervention will involve aerobic and resistance exercise during 3 phases of the ASCT pathway:

- Phase 1 (6-8 weeks before hospital admission for SCT): Participants will receive one exercise session per week, supervised by a physiotherapist, at a UCLH gym. Participants will be requested to complete two further sessions per week independently.
- Phase 2: (during hospital admission): Immediately after SCT, participants will be offered a supervised exercise session with a physiotherapist in the hospital setting three times per week.

For peer review only - http://bmjopen.bmj.com/site/about/guidelines.xhtml

- Phase 3 (12 weeks after hospital discharge): Participants will be asked to continue exercising independently three times per week and will receive a weekly phone call from a physiotherapist for support and guidance.

Supervised aerobic exercise will comprise of treadmill walking or stationary cycling. Participants will be encouraged to use walking as their aerobic exercise activity, or a stationary bike or elliptical trainer if they have access to one, for independent exercise sessions. In phase 1, prior to transplant admission, aerobic exercise intensity will be targeted between 60-80% of heart rate (HR) reserve (or Karvonen formula), in which Target HR = [(max HR − resting HR) × % intensity] + resting HR, where maximum HR = 220-age[26]. Duration will be started at 15 minutes and progressed by 5 minutes per week to achieve a minimum of 30 minutes by week 3-4. This will be further progressed to 40 minutes by week 5. Target HR will be monitored using HR belts and participants will also use a Rating of Perceived Exertion Scale (RPE)[27]. The participants will be given a scale in their exercise log book, instructed in its use, and advised to work to levels of exertion as determined under supervision. Participants will be encouraged to reach the target duration in one bout but in the presence of discomfort or wish to change exercise machine/method then participants will be required to complete the target duration in bouts of 15 minutes or more.

Resistance exercises will be prescribed to target all major muscle groups (depending on individual health and contraindications). Resistance training will be performed using body weight, progressed with weights or using elastic resistance bands of various strengths. These bands will be supplied to participants at the start of the study to allow convenient independent resistance training. Starting resistance and repetitions of the exercises will be informed by a 10-repetition maximum assessment carried out by the physiotherapist during the first session. Individually tailoring and gradual progression in resistance training will be prescribed as deemed appropriate at each exercise session by the physiotherapist according to published principles[28]. Participants will be provided with

For peer review only - http://bmjopen.bmj.com/site/about/guidelines.xhtml

For peer review only

exercise log books and asked to record what exercise they do in the books, as well as document how they feel after exercising and any reasons for not carrying out the exercise as prescribed.

In phase 2, when participants are admitted to hospital for their ASCT, they will be offered supervised exercise with the physiotherapist three times per week in the ambulatory care or hospital ward setting (depending on each participant's clinical location), which they can decline if they wish to do so. It is anticipated that participants will be symptomatic from the effects of their ASCT, which may include nausea, gastrointestinal complaints and fatigue. They may also experience bleeding and infection as a result of thrombocytopenia and low white cell counts. Therefore the exercise intervention will be highly individualised and tailored according to participant's capacity to carry out the exercises. Aerobic exercise will be carried out, as able, on a stationary bike in bouts of 10 minutes, up to a total duration of 30 minutes. HR will be monitored throughout and participants will be guided to exercise at a RPE (determined by RPE scale) they are comfortable with. Any exercise undertaken, declined or not possible due to clinical indication will be recorded by the physiotherapist in the participants' log book.

In phase 3, when the participants have been discharged from hospital they will be advised by the physiotherapist to continue the independent exercise programme, tailored to their fitness on discharge, until they are 12 weeks post-ASCT. During these 12 weeks, they will be contacted once per week via telephone by the physiotherapist to provide support and guidance with continuing to exercise regularly during their recovery and to assist them in progressing their exercise programme. The participants will be encouraged to meet recommended guidance for levels of PA[29] and requested to record any exercise undertaken in their log books.

Previous systematic reviews and meta-analyses have shown that behaviour change techniques (BCTs) from exercise interventions that are associated with improved adherence to exercise by cancer survivors include 'goal setting (behaviour)', setting 'graded tasks', and 'instruction of how to perform

For peer review only - http://bmjopen.bmj.com/site/about/guidelines.xhtml

BCT Label	BCT No. (BCTT v1)	Component of Intervention
Goal setting (behaviour)	1.1	Overarching goal of exercise programme for all participants is to exercise 3 times per week. Participants also supported to define own personal sub-goals, which are recorded in log book.
Problem Solving	1.2	Enablers, barriers and solutions to barriers completed with physiotherapist and recorded in log book. Problem solving with physiotherapist if discrepancy between current behaviour and goal is identified.
Action Planning	1.4	Attendance at supervised exercise session in gym planned for specific day each week. Participants are also supported to plan independent exercise sessions (e.g. time/day of the week) with physiotherapist. These plans are recorded in log book prior to planned execution.
Review Behaviour Goal	1.5	Participants review performance of exercise sessions recorded in log book against planned goals, and consider modifying goals accordingly with physiotherapist in supervised exercise

behaviour'[30,31] practice and self-monitoring and encouraging participants to attempt to generalise exercise behaviours learned in supervised exercise environments to other, non-supervised contexts[32]. Strategies to promote adherence to the intervention and promote change in exercise behaviour in the current intervention have been coded according to the BCT Taxonomy v1[33]. See Table 1 for incorporated BCTs and examples of how they have been included in the intervention. Physiotherapists who deliver the intervention will receive training, clinical supervision and will be provided with a standard operating procedure to carry out the intervention and support the participants to exercise and using BCTs.

For peer review only

		sessions.
Discrepancy between current behaviour and goal	1.6	Physiotherapist informs participant if there is a discrepancy between current behaviour and goal.
Feedback on behaviour	2.2	Feedback on performance of exercise programme delivered by physiotherapist during supervised exercise sessions (phase 1 and 2) and telephone calls (phase 3).
Self-monitoring of behaviour	2.3	Participants asked to record exercise carried out each week in log book. Heart rate monitoring used in supervised and independent exercise sessions to monitor behaviour being carried out at target intensity.
Biofeedback	2.6	Heart rate monitoring of aerobic exercise effort.
Instruction on how to perform the behaviour	4.1	Supervised exercise sessions with physiotherapist who instructs/teaches participant how to perform behaviour.
Information about health consequences	5.1	Physiotherapist and log book provides information about effects of exercise for health in context of myeloma.
Monitoring of emotional consequences	5.4	Participants encouraged to complete weekly reflections in log book.
Information about emotional consequences	5.6	Physiotherapist and log book provide information about effects of exercise on emotional wellbeing.
Demonstration of the behaviour	6.1	Physiotherapist demonstration and teaching of exercise programme in supervised sessions.
Behavioural practice/rehearsal	8.1	Exercises taught/demonstrated in supervised sessions are practiced within those sessions and independent sessions (phase 1 and 2).
Generalisation of target behaviour	8.6	Advice to perform exercise programme, learned in supervised session, in independent sessions, during hospital admission and post discharge.
Graded tasks	8.7	Progression of exercise sessions throughout exercise period.
Credible source	9.1	Education and prompting from a physiotherapist

For peer review only - http://bmjopen.bmj.com/site/about/guidelines.xhtml

		with expertise in myeloma/haematology and provided in log book.
Pros and cons	9.2	Prompts to consider advantages and disadvantages of exercising through discussion with physiotherapist and recording in log book.
Adding objects to the environment	12.5	Provision of heart rate monitors, resistance exercise bands and exercise programme sheets/log book for use in independent sessions.
Verbal persuasion about capability	15.1	Verbal support and supervision of physiotherapist to encourage exercise sessions before, during and after transplant treatment.

Table 1: Behaviour Change Techniques (BCT) coded to BCT Taxonomy (BCTT v1) (Michie *et al.*, 2013)

For peer review only

Usual care (control) group

Participants randomised to usual care will receive the usual advice provided by haematology CNSs. Participants who specifically ask the research team for exercise advice will be signposted to generic PA advice offered for people undergoing cancer treatment on the Macmillan Cancer support website[34] or from the information and support service within UCLH.

During hospital admission, patients undergoing SCT are routinely screened by a hospital physiotherapist and receive input for any functional or mobility-related deficits that may prevent or delay hospital discharge. All patients within the study who require physiotherapy or occupational therapy during their hospital admission will receive input as indicated. The indication for and details of input required will be obtained from participants' hospital records and recorded in the study file for enrolled participants who require additional therapy input. Control group participants will not be asked to monitor their activity or receive log books.

Outcome Assessment

The primary endpoints of this pilot study will be study feasibility. A target recruitment rate of >50% of potential participants screened as eligible and approached for this study will be a primary indication of feasibility. Feasibility of delivering the intervention protocol will be assessed by evaluating adherence to the intervention and acceptability of receiving the intervention (including deviations from exercise protocol). Attrition due to the intervention, loss to follow-up and adverse events will also be assessed.

For peer review only - http://bmjopen.bmj.com/site/about/guidelines.xhtml

A number of secondary endpoints will also evaluate any between group differences and determine intended primary and secondary endpoints for a fully-powered trial. Fatigue will be assessed using the Functional Assessment of Chronic Illness Therapy (FACIT-F) questionnaire[35]. QOL will be measured with two complementary instruments; the European Organisation for Research and Treatment of Cancer QOL Questionnaire (EORTC QLQ-C30 and myeloma specific module QLQMY20)[36,37] and the Functional Assessment of Cancer Therapy Bone Marrow Transplantation scale (FACT-BMT) questionnaire[38]. The EORTC QLQ C-30 has a focus on physical and functioning dimensions of QOL whereas FACT-BMT can assess impact of treatment and side-effects upon QOL. Comparison analysis indicates that both instruments should be used together rather than in place of each other when assessing QOL in patients undergoing SCT[39,40].

Functional capacity will be assessed with a six-minute walk test (6MWT), hand-held dynamometry and a timed sit to stand test. PA and sedentary time will be recorded over 5-7 days using an ActivPAL accelerometer (PAL Technologies Ltd. Glasgow, UK). Additional measures of self-reported PA behaviour will be captured using the self-complete short form International Physical Activity Questionnaire (IPAQ-SF)[41] and the Exercise Self-Efficacy Scale (ESES)[42]. All these measures will be recorded at baseline and three time points during the ASCT pathway. At the final time point, an adapted Client Service Receipt Inventory questionnaire will be used to assess health and social care usage in the time following hospital discharge[43].

The outcomes measures and time points for data collection are summarised in table 2.

For peer review only

	Screening	Baseline Assessment (T0)	Follow up 1 (T1)	Follow up 2 (T2)	Follow up 3 (T3)
Visit No:		1	2	3	4
	On referral to UCLH ASCT MDT	On day of first transplant clinic appointment	Day of transplant	Day of discharge from hospital	Post-transplant follow-up clinic at 3 months
Qualitative Interview (~20 patients who decline study)	X				
Window of flexibility for timing of visits:		+/-2 days	0+/-1 day	0+/-1 day	+/- 5 days
Screening eligibility	X				
Informed Consent		X			
Demographics & Clinical History		X			
Fatigue: FACT-F		X	X	X	X
QOL: EORTC QLQ C-30-MY20		X	X	X	X
QOL: FACT-BMT		X	X	X	X
Exercise Behaviour: IPAQ-SF		X	X	X	X
Exercise Self-efficacy Scale		X	X	X	X
Functional Capacity: 6MWT		X	X	X	X
Functional Capacity: 30s STS		X	X	X	X
Functional Capacity: hand held dynamometry		X	X	X	X
Resting BP and HR		X	X	X	X
Height and weight		X	X	X	X
Accelerometery (3-7 days)		X	X	X	X
Qualitative Interview (20 participants)					X
Health and Social Care Service Use: CRSI					X
Blood Counts		X	X	X	X
Levels of Immune Function (blood)		X	X	X	X

For peer review only

Table 2: Study schedule of assessments
Table abbreviations: UCLH, University College London Hospitals NHS Foundation Trust. ASCT, autologous stem cell transplant. MDT, multidisciplinary team. FACT-F, Functional Assessment of Chronic Illness Therapy Fatigue questionnaire. EORTC QLQ C-30-MY20, European Organisation for Research and Treatment of Cancer QOL Questionnaire and myeloma specific module. FACT-BMT, Functional Assessment of Cancer Therapy Bone Marrow Transplantation scale. IPAQ-SF, International Physical Activity Questionnaire short form. 6MWT, six minute walk test. STS, sit to stand. BP, blood pressure. HR, heart rate. CRSI, Client Service Receipt Inventory.

For peer review only - http://bmjopen.bmj.com/site/about/guidelines.xhtml

Qualitative Data:

Semi-structured interviews will be conducted with a purposeful sample of participants at two time points. Study 'decliners', deemed eligible for the study, who have received a study PIS but declined to enrol in the study will be asked if they would participate in a short interview. These interviews will take place either face to face in the clinic setting or via telephone (based on participants' preference) and will be conducted to explore the patients' experiences of being approached for the exercise study and the process and outcome of their decision-making process in relation to the exercise study.

Additional semi-structured interviews from approximately 20 participants who enrol and take part in the study will take place at final follow-up, approximately 3 months post-transplant, and will be conducted to explore the participants experiences of ASCT treatment and their experiences of being enrolled in the study. Participants will be purposively sampled to include participants from both the intervention arm and the control arm.

Anticipated dates of trial commencement and completion

Recruitment commenced in June 2019, with data collection for all follow-up assessments estimated to be completed by December 2020.

Statistical analysis including sample size calculations

This is a pilot study with no formal power calculation. There is limited available data on the application of an exercise intervention delivered prior to ASCT in people with myeloma. This study will provide estimates of means/standard deviations for the intended outcomes of a fully-powered RCT, to support a sample size calculation for a larger trial. Meaningful clinically important differences are known for a number of the secondary endpoints, including the FACIT-F[44], EORTC-QLQ-30 and MY20 module[45,46] and 6MWT[47] and will be used to inform future sample size calculations. For a pilot study, including >30 participants in each arm is considered to be an acceptable number[48,49].

For peer review only - http://bmjopen.bmj.com/site/about/guidelines.xhtml

For peer review only

Data analysis

The primary outcomes (intervention recruitment rate and intervention adherence) from the trial will be summarised as percentages or mean values for each item, with 95% confidence intervals. Data on the acceptability of the randomisation through its effect on dropout rates, completion rates of the outcome assessments and reported use and satisfaction with the intervention materials will also be analysed descriptively. Group differences in fatigue, QOL, functional capacity, PA and sedentary time, self-efficacy at each time point will be analysed using linear regression for continuous factors (e.g. fatigue) and frequency tables, chi-squared tests and logistic regression for categorical factors. Gender and age, will be included within the models as covariates. Repeated measures analyses (e.g. mixed models) will be used to analyse the baseline and follow up scores of all measures together. Assessments for each outcome will be made to determine whether the data are normally distributed. For outcomes that are not, even after appropriate transformations, non-parametric methods will be used for data analyses at specific time points.

Qualitative data derived from the interviews of patients will be analysed thematically using an approach that is both deductive and inductive, to ensure that the full range of participants' responses are represented. Codes generated from the data will be assigned to portions of the text to develop an initial coding framework, which will be used by another researcher familiar with the sample population to independently double-code a proportion of the interview transcripts. The framework will be revised to ensure that the codes accurately reflect the data and any discrepancies will be resolved via discussion. The final codes will be applied to the data and incorporated into appropriate themes/sub-themes.

ETHICS & DISSEMINATION

For peer review only - http://bmjopen.bmj.com/site/about/guidelines.xhtml

This study has been approved by the NHS Health Authority Research Ethics Committee – Camden and Kings Cross – reference 19/LO/0204 (Protocol Version 1.0 14/12/2018). All participants will provide informed written consent to take part and can withdraw at any time.

Results of this study will be written up as part of a doctoral thesis and disseminated through publication in peer reviewed journals, presentation at haematology and rehabilitation-related meetings and shared locally. A lay summary of the study results will be provided to participants who request to receive one after the study have completed.

For peer review only

DISCUSSION

The study described in this protocol will evaluate the feasibility of carrying out an exercise intervention among people with myeloma before, during and after autologous SCT at a UK centre. There is no standardised or accepted approach to using lead time before SCT to prepare and physically optimise patients and the importance of carrying out research of this kind is particularly pertinent in this population for whom SCT currently offers the best possibility of longer progression-free survival, yet carries the risk of considerable morbidity.

The strengths of this study include the use of a pragmatic intervention using BCTs and designed to integrate into the existing ASCT treatment pathway at our centre and the use of a partly-supervised exercise intervention among myeloma patients preparing for transplant. This study is also the first of its kind in the UK to incorporate a control arm. If the feasibility criteria for trial is met and this single site, pilot trial is deemed to be successful it is anticipated to be an easily replicable trial design for future evaluation as a fully-powered multicentre trial. Recent guidance for prehabilitation in cancer advocates for a multimodal intervention, comprising of exercise, nutrition and psychological/behaviour change assessment and support[50]. There is no nutritional assessment or dietary intervention included in this study which may be considered a limitation of this exercise

For peer review only - http://bmjopen.bmj.com/site/about/guidelines.xhtml

focussed intervention. It is expected that this pilot study will be acceptable and feasible and an important step in developing the field of exercise prehabilitation and rehabilitation within haematological cancer treatment in the UK. Dissemination of study results is expected in early 2021.

AUTHORS CONTRIBUTIONS

OM drafted the manuscript. OM, KY, AF, GR designed the study and secured funding and ethical approval. AR, JL, NR provided intellectual input to the final study protocol according to their area of expertise. All authors contributed to revising and final approval of the submitted manuscript.

FUNDING STATEMENT

This report is independent research supported by the National Institute for Health Research (HEE/ NIHR ICA Programme Clinical Doctoral Research Fellowship, Ms Orla McCourt, ICA-CDRF-2017-03-067). The views expressed in this publication are those of the author(s) and not necessarily those of the NHS, the National Institute for Health Research or the Department of Health and Social Care.

COMPETING INTERESTS STATEMENT

None

For peer review only

References:

1. Haematological Malignancy Research Network (HMRN). Prevalence 2019. Available from: https://www.hmrn.org/statistics/prevalence [Accessed June 2019].

2. Gozzetti A, Candi V, Papini G, et al. Therapeutic Advancements in Multiple Myeloma. *Frontiers in Oncology* 2014;4:241.

3. Myeloma UK. Symptoms and Complications. Available from: https://www.myeloma.org.uk/information/symptoms-and-complications/ [Accessed 18th April].

4. Cancer Research UK. Myeloma Statistics 2015. Available from: http://www.cancerresearchuk.org/health-professional/cancer-statistics/statistics-by-cancer-type/myeloma#heading-Zero [Accessed June 2019].

5. Mahajan S, Tandon N, Kumar S. The evolution of stem-cell transplantation in multiple myeloma. *Ther Adv Hematol* 2018;9(5):123-33.

6. NHS Commissioning Board. Clinical Commissioning Policy: Haematopoietic Stem Cell Transplantation England: NHS Commissioning Board; 2013.

7. Krejci M, Scudla V, Tothova E, et al. Long-term outcomes of autologous transplantation in multiple myeloma: significant survival benefit of novel drugs in post-transplantation relapse. *Clin Lymphoma Myeloma* 2009;9(6):436-42.

8. NHS England. Clinical Commissioning Policy: Haematolopoietic Stem Cell Transplantation (HSCT): Revised (All Ages). In: England N, editor. England2015.

9. British Society of Blood and Marrow Transplantation. 2016 Activity 2016. Available from: http://www.bsbmt.org/activity/2016/ [Accessed June 2019].

10. Pidala J, Anasetti C, Jim H. Health-related quality of life following haematopoietic cell transplantation: patient education, evaluation and intervention. *Br J Haematol* 2010;148(3):373-85.

11. Ahmedzai SH, Snowden JA, Ashcroft AJ, et al. Patient-Reported Outcome Results From the Open-Label, Randomized Phase III Myeloma X Trial Evaluating Salvage Autologous Stem-Cell Transplantation in Relapsed Multiple Myeloma. *Journal of clinical oncology : official journal of the American Society of Clinical Oncology* 2019:Jco1801006.

12. Snowden JA, Greenfield DM, Bird JM, et al. Guidelines for screening and management of late and long-term consequences of myeloma and its treatment. *Br J Haematol* 2017;176(6):888-907.

13. Craike MJ, Hose K, Courneya KS, et al. Perceived benefits and barriers to exercise for recently treated patients with multiple myeloma: a qualitative study. *BMC cancer* 2013;13:319-.

14. Liu RD, Chinapaw MJ, Huijgens PC, et al. Physical exercise interventions in haematological cancer patients, feasible to conduct but effectiveness to be established: a systematic literature review. *Cancer Treat Rev* 2009;35(2):185-92.

For peer review only - http://bmjopen.bmj.com/site/about/guidelines.xhtml

15. Wiskemann J, Huber G. Physical exercise as adjuvant therapy for patients undergoing hematopoietic stem cell transplantation. *Bone marrow transplantation* 2008;41(4):321-9.

16. Persoon S, Kersten MJ, van der Weiden K, et al. Effects of exercise in patients treated with stem cell transplantation for a hematologic malignancy: a systematic review and meta-analysis. *Cancer Treat Rev* 2013;39(6):682-90.

17. van Haren IE, Timmerman H, Potting CM, et al. Physical exercise for patients undergoing hematopoietic stem cell transplantation: systematic review and meta-analyses of randomized controlled trials. *Phys Ther* 2013;93(4):514-28.

18. Cohen MZ, Rozmus CL, Mendoza TR, et al. Symptoms and quality of life in diverse patients undergoing hematopoietic stem cell transplantation. *Journal of pain and symptom management* 2012;44(2):168-80.

19. Yin X, Tang L, Fan F, et al. Allogeneic stem-cell transplantation for multiple myeloma: a systematic review and meta-analysis from 2007 to 2017. *Cancer Cell Int* 2018;18:62.

20. Coleman EA, Coon S, Hall-Barrow J, et al. Feasibility of exercise during treatment for multiple myeloma. *Cancer nursing* 2003;26(5):410-9.

21. Coleman EA, Coon SK, Kennedy RL, et al. Effects of exercise in combination with epoetin alfa during high-dose chemotherapy and autologous peripheral blood stem cell transplantation for multiple myeloma. *Oncol Nurs Forum* 2008;35(3):E53-61.

22. Coleman EA, Goodwin JA, Kennedy R, et al. Effects of exercise on fatigue, sleep, and performance: a randomized trial. *Oncology nursing forum* 2012;39(5):468-77.

23. Groeneveldt L, Mein G, Garrod R, et al. A mixed exercise training programme is feasible and safe and may improve quality of life and muscle strength in multiple myeloma survivors. *BMC cancer* 2013;13:31.

24. Keen C, Skilbeck J, Ross H, et al. Is it feasible to conduct a randomised controlled trial of pretransplant exercise (prehabilitation) for patients with multiple myeloma awaiting autologous haematopoietic stem cell transplantation? Protocol for the PREeMPT study. *BMJ Open* 2018;8(3).

25. Macmillan Cancer Support. Impact Briefs: Cancer Clinical Nurse Specialists 2012. Available from: https://www.macmillan.org.uk/documents/aboutus/commissioners/clinicalnursespecialistsanevidencereview2012.pdf [Accessed November 2019].

26. Karvonen MJ, Kentala E, Mustala O. The effects of training on heart rate; a longitudinal study. *Annales medicinae experimentalis et biologiae Fenniae* 1957;35(3):307-15.

27. Borg GA. Psychophysical bases of perceived exertion. *Med Sci Sports Exerc* 1982;14(5):377-81.

28. Kraemer WJ, Ratamess NA. Fundamentals of resistance training: progression and exercise prescription. *Med Sci Sports Exerc* 2004;36(4):674-88.

For peer review only - http://bmjopen.bmj.com/site/about/guidelines.xhtml

29. Department of Health & Social Care. Start active, stay active: a report of physical activity from the four home countries Chief Medical Officers. 2011.

30. Bourke L, Homer KE, Thaha MA, et al. Interventions for promoting habitual exercise in people living with and beyond cancer. *Cochrane Database Syst Rev* 2013(9):CD010192.

31. Turner RR, Steed L, Quirk H, et al. Interventions for promoting habitual exercise in people living with and beyond cancer. *Cochrane Database Syst Rev* 2018;9:Cd010192.

32. Hobbs N, Godfrey A, Lara J, et al. Are behavioral interventions effective in increasing physical activity at 12 to 36 months in adults aged 55 to 70 years? a systematic review and meta-analysis. *BMC Medicine* 2013;11(1):75.

33. Michie S, Richardson M, Johnston M, et al. The behavior change technique taxonomy (v1) of 93 hierarchically clustered techniques: building an international consensus for the reporting of behavior change interventions. *Ann Behav Med* 2013;46(1):81-95.

34. Macmillan Cancer Support. Physical Activity Resources 2019. Available from: https://www.macmillan.org.uk/about-us/health-professionals/programmes-and-services/physical-activity/resources.html [Accessed July 2019].

35. Webster K, Cella D, Yost K. The Functional Assessment of Chronic Illness Therapy (FACIT) Measurement System: properties, applications, and interpretation. *Health Qual Life Outcomes* 2003;1:79.

36. Aaronson NK, Ahmedzai S, Bergman B, et al. The European Organization for Research and Treatment of Cancer QLQ-C30: a quality-of-life instrument for use in international clinical trials in oncology. *J Natl Cancer Inst* 1993;85(5):365-76.

37. Cocks K, Cohen D, Wisloff F, et al. An international field study of the reliability and validity of a disease-specific questionnaire module (the QLQ-MY20) in assessing the quality of life of patients with multiple myeloma. *Eur J Cancer* 2007;43(11):1670-8.

38. McQuellon RP, Russell GB, Cella DF, et al. Quality of life measurement in bone marrow transplantation: development of the Functional Assessment of Cancer Therapy-Bone Marrow Transplant (FACT-BMT) scale. *Bone marrow transplantation* 1997;19(4):357-68.

39. Grulke N, Albani C, Bailer H. Quality of life in patients before and after haematopoietic stem cell transplantation measured with the European Organization for Research and Treatment of Cancer (EORTC) Quality of Life Core Questionnaire QLQ-C30. *Bone marrow transplantation* 2012;47(4):473-82.

40. Kopp M, Schweigkofler H, Holzner B, et al. EORTC QLQ-C30 and FACT-BMT for the measurement of quality of life in bone marrow transplant recipients: a comparison. *European journal of haematology* 2000;65(2):97-103.

For peer review only - http://bmjopen.bmj.com/site/about/guidelines.xhtml

41. Craig CL, Marshall AL, Sjostrom M, et al. International physical activity questionnaire: 12-country reliability and validity. *Med Sci Sports Exerc* 2003;35(8):1381-95.

42. Resnick B, Jenkins LS. Testing the reliability and validity of the Self-Efficacy for Exercise scale. *Nurs Res* 2000;49(3):154-9.

43. Beecham J, Knapp, M. Costing psychiatric interventions. In: Thornicroft G, editor. Measuring Mental Health Needs. 2nd ed: Gaskell; 2001. p. 200-24.

44. Cella D, Yount S, Sorensen M, et al. Validation of the Functional Assessment of Chronic Illness Therapy Fatigue Scale relative to other instrumentation in patients with rheumatoid arthritis. *J Rheumatol* 2005;32(5):811-9.

45. Kvam AK, Wisloff F, Fayers PM. Minimal important differences and response shift in health-related quality of life; a longitudinal study in patients with multiple myeloma. *Health Qual Life Outcomes* 2010;8:79.

46. Sully K, Trigg A, Bonner N, et al. Estimation of minimally important differences and responder definitions for EORTC QLQ-MY20 scores in multiple myeloma patients. *European journal of haematology* 2019;103(5):500-9.

47. Bohannon RW, Crouch R. Minimal clinically important difference for change in 6-minute walk test distance of adults with pathology: a systematic review. *J Eval Clin Pract* 2017;23(2):377-81.

48. Lancaster GA, Dodd S, Williamson PR. Design and analysis of pilot studies: recommendations for good practice. *J Eval Clin Pract* 2004;10(2):307-12.

49. Browne RH. On the use of a pilot sample for sample size determination. *Stat Med* 1995;14(17):1933-40.

50. Macmillan Cancer Support. Prehabilitation for people with cancer: Principles and guidance for prehabilitation within the management and support of people with cancer. England; 2019.

For peer review only

For peer review only - http://bmjopen.bmj.com/site/about/guidelines.xhtml

STANDARD PROTOCOL ITEMS: RECOMMENDATIONS FOR INTERVENTIONAL TRIALS

SPIRIT 2013 Checklist: Recommended items to address in a clinical trial protocol and related documents*

Section/item	Item No	Description	Addressed on page number
**Administrative information**			
Title	1	Descriptive title identifying the study design, population, interventions, and, if applicable, trial acronym	1
Trial registration	2a	Trial identifier and registry name. If not yet registered, name of intended registry	3
	2b	All items from the World Health Organization Trial Registration Data Set	______________
Protocol version	3	Date and version identifier	20 – line 364
Funding	4	Sources and types of financial, material, and other support	21 – line 397
Roles and responsibilities	5a	Names, affiliations, and roles of protocol contributors	1; 21 – line 392
	5b	Name and contact information for the trial sponsor	______________
	5c	Role of study sponsor and funders, if any, in study design; collection, management, analysis, and interpretation of data; writing of the report; and the decision to submit the report for publication, including whether they will have ultimate authority over any of these activities	21 – line 397
	5d	Composition, roles, and responsibilities of the coordinating centre, steering committee, endpoint adjudication committee, data management team, and other individuals or groups overseeing the trial, if applicable (see Item 21a for data monitoring committee)	______________

For peer review only

Introduction

Background and rationale	6a	Description of research question and justification for undertaking the trial, including summary of relevant studies (published and unpublished) examining benefits and harms for each intervention	4-6 - lines 46-100
	6b	Explanation for choice of comparators	______________
Objectives	7	Specific objectives or hypotheses	6 – lines 102-119
Trial design	8	Description of trial design including type of trial (eg, parallel group, crossover, factorial, single group), allocation ratio, and framework (eg, superiority, equivalence, noninferiority, exploratory)	6 – line 103

Methods: Participants, interventions, and outcomes

Study setting	9	Description of study settings (eg, community clinic, academic hospital) and list of countries where data will be collected. Reference to where list of study sites can be obtained	8 – lines 150-154
Eligibility criteria	10	Inclusion and exclusion criteria for participants. If applicable, eligibility criteria for study centres and individuals who will perform the interventions (eg, surgeons, psychotherapists)	7 – lines 130-140
Interventions	11a	Interventions for each group with sufficient detail to allow replication, including how and when they will be administered	10 – lines 184-272
	11b	Criteria for discontinuing or modifying allocated interventions for a given trial participant (eg, drug dose change in response to harms, participant request, or improving/worsening disease)	10-11 – lines 202-211
	11c	Strategies to improve adherence to intervention protocols, and any procedures for monitoring adherence (eg, drug tablet return, laboratory tests)	12 – lines 245-255
	11d	Relevant concomitant care and interventions that are permitted or prohibited during the trial	15 – lines 266-272
Outcomes	12	Primary, secondary, and other outcomes, including the specific measurement variable (eg, systolic blood pressure), analysis metric (eg, change from baseline, final value, time to event), method of aggregation (eg, median, proportion), and time point for each outcome. Explanation of the clinical relevance of chosen efficacy and harm outcomes is strongly recommended	15-26 – lines 274-301
Participant timeline	13	Time schedule of enrolment, interventions (including any run-ins and washouts), assessments, and visits for participants. A schematic diagram is highly recommended (see Figure)	17

For peer review only - http://bmjopen.bmj.com/site/about/guidelines.xhtml

Sample size	14	Estimated number of participants needed to achieve study objectives and how it was determined, including clinical and statistical assumptions supporting any sample size calculations	9 – lines 169-171
Recruitment	15	Strategies for achieving adequate participant enrolment to reach target sample size	8 – lines 156-165
**Methods: Assignment of interventions (for controlled trials)**			
Allocation:			
Sequence generation	16a	Method of generating the allocation sequence (eg, computer-generated random numbers), and list of any factors for stratification. To reduce predictability of a random sequence, details of any planned restriction (eg, blocking) should be provided in a separate document that is unavailable to those who enrol participants or assign interventions	9 – lines 175-178
Allocation concealment mechanism	16b	Mechanism of implementing the allocation sequence (eg, central telephone; sequentially numbered, opaque, sealed envelopes), describing any steps to conceal the sequence until interventions are assigned	9 – lines 179-182
Implementation	16c	Who will generate the allocation sequence, who will enrol participants, and who will assign participants to interventions	______________
Blinding (masking)	17a	Who will be blinded after assignment to interventions (eg, trial participants, care providers, outcome assessors, data analysts), and how	10 – lines 181-182
	17b	If blinded, circumstances under which unblinding is permissible, and procedure for revealing a participant's allocated intervention during the trial	______________
**Methods: Data collection, management, and analysis**			
Data collection methods	18a	Plans for assessment and collection of outcome, baseline, and other trial data, including any related processes to promote data quality (eg, duplicate measurements, training of assessors) and a description of study instruments (eg, questionnaires, laboratory tests) along with their reliability and validity, if known. Reference to where data collection forms can be found, if not in the protocol	15 – lines 282-301
	18b	Plans to promote participant retention and complete follow-up, including list of any outcome data to be collected for participants who discontinue or deviate from intervention protocols	18 – lines 314-318

Data management	19	Plans for data entry, coding, security, and storage, including any related processes to promote data quality (eg, double data entry; range checks for data values). Reference to where details of data management procedures can be found, if not in the protocol	______________
Statistical methods	20a	Statistical methods for analysing primary and secondary outcomes. Reference to where other details of the statistical analysis plan can be found, if not in the protocol	18 – lines 330-337
	20b	Methods for any additional analyses (eg, subgroup and adjusted analyses)	19 – lines 339-360
	20c	Definition of analysis population relating to protocol non-adherence (eg, as randomised analysis), and any statistical methods to handle missing data (eg, multiple imputation)	______________
**Methods: Monitoring**			
Data monitoring	21a	Composition of data monitoring committee (DMC); summary of its role and reporting structure; statement of whether it is independent from the sponsor and competing interests; and reference to where further details about its charter can be found, if not in the protocol. Alternatively, an explanation of why a DMC is not needed	______________
	21b	Description of any interim analyses and stopping guidelines, including who will have access to these interim results and make the final decision to terminate the trial	______________
Harms	22	Plans for collecting, assessing, reporting, and managing solicited and spontaneously reported adverse events and other unintended effects of trial interventions or trial conduct	15 – lines 279-280
Auditing	23	Frequency and procedures for auditing trial conduct, if any, and whether the process will be independent from investigators and the sponsor	______________
**Ethics and dissemination**			
Research ethics approval	24	Plans for seeking research ethics committee/institutional review board (REC/IRB) approval	20 – lines 362-369
Protocol amendments	25	Plans for communicating important protocol modifications (eg, changes to eligibility criteria, outcomes, analyses) to relevant parties (eg, investigators, REC/IRBs, trial participants, trial registries, journals, regulators)	______________

For peer review only - http://bmjopen.bmj.com/site/about/guidelines.xhtml

Consent or assent	26a	Who will obtain informed consent or assent from potential trial participants or authorised surrogates, and how (see Item 32)	8 – lines 161-165
	26b	Additional consent provisions for collection and use of participant data and biological specimens in ancillary studies, if applicable	______________
Confidentiality	27	How personal information about potential and enrolled participants will be collected, shared, and maintained in order to protect confidentiality before, during, and after the trial	______________
Declaration of interests	28	Financial and other competing interests for principal investigators for the overall trial and each study site	21 – line 402
Access to data	29	Statement of who will have access to the final trial dataset, and disclosure of contractual agreements that limit such access for investigators	______________
Ancillary and post-trial care	30	Provisions, if any, for ancillary and post-trial care, and for compensation to those who suffer harm from trial participation	______________
Dissemination policy	31a	Plans for investigators and sponsor to communicate trial results to participants, healthcare professionals, the public, and other relevant groups (eg, via publication, reporting in results databases, or other data sharing arrangements), including any publication restrictions	20 – lines 366-369
	31b	Authorship eligibility guidelines and any intended use of professional writers	______________
	31c	Plans, if any, for granting public access to the full protocol, participant-level dataset, and statistical code	______________
**Appendices**			
Informed consent materials	32	Model consent form and other related documentation given to participants and authorised surrogates	______________
Biological specimens	33	Plans for collection, laboratory evaluation, and storage of biological specimens for genetic or molecular analysis in the current trial and for future use in ancillary studies, if applicable	______________

*It is strongly recommended that this checklist be read in conjunction with the SPIRIT 2013 Explanation & Elaboration for important clarification on the items. Amendments to the protocol should be tracked and dated. The SPIRIT checklist is copyrighted by the SPIRIT Group under the Creative Commons "Attribution-NonCommercial-NoDerivs 3.0 Unported" license.

For peer review only
